# Adipose-Derived Stem Cells Stimulate Regeneration of Peripheral Nerves: BDNF Secreted by These Cells Promotes Nerve Healing and Axon Growth *De Novo*

**DOI:** 10.1371/journal.pone.0017899

**Published:** 2011-03-14

**Authors:** Tatiana Lopatina, Natalia Kalinina, Maxim Karagyaur, Dmitry Stambolsky, Kseniya Rubina, Alexander Revischin, Galina Pavlova, Yelena Parfyonova, Vsevolod Tkachuk

**Affiliations:** 1 Department of Biochemistry and Molecular Medicine, Faculty of Fundamental Medicine, Lomonosov Moscow State University, Moscow, Russia; 2 Institute of Gene Biology, Russian Academy of Sciences, Moscow, Russia; Université de Technologie de Compiègne, France

## Abstract

Transplantation of adipose-derived mesenchymal stem cells (ASCs) induces tissue regeneration by accelerating the growth of blood vessels and nerve. However, mechanisms by which they accelerate the growth of nerve fibers are only partially understood. We used transplantation of ASCs with subcutaneous matrigel implants (well-known in vivo model of angiogenesis) and model of mice limb reinnervation to check the influence of ASC on nerve growth. Here we show that ASCs stimulate the regeneration of nerves in innervated mice's limbs and induce axon growth in subcutaneous matrigel implants. To investigate the mechanism of this action we analyzed different properties of these cells and showed that they express numerous genes of neurotrophins and extracellular matrix proteins required for the nerve growth and myelination. Induction of neural differentiation of ASCs enhances production of brain-derived neurotrophic factor (BDNF) as well as ability of these cells to induce nerve fiber growth. BDNF neutralizing antibodies abrogated the stimulatory effects of ASCs on the growth of nerve sprouts. These data suggest that ASCs induce nerve repair and growth via BDNF production. This stimulatory effect can be further enhanced by culturing the cells in neural differentiation medium prior to transplantation.

## Introduction

Recently, cell therapy has been proposed as an efficient method for regenerating injured nerves [1]. Transplantation of Schwann cells or stem cells of various origins, which differentiate towards Schwann cell-like phenotype, stimulate peripheral nerve repair. Transplanted cells stimulate the growth and myelination of nerve sprouts by secreting neurotrophins and neuroregulins together with components of myelin shell [2].

However, obtaining Schwann cells for autologous transplantation is highly traumatic and these cells are difficult to expand in vitro [3]. Therefore, there is a need for a more easily accessible source of cells that are capable of stimulating nerve sprout growth and repair. Adipose-derived stem cells (ASCs) can be easily obtained and expanded in vitro for use in autologous cell therapy. Thus, transplanted ASCs stimulate blood vessel growth *in vivo* [4], [5]. This effect is dependent on the secretion of growth factors, VEGF, HGF and bFGF, and enhanced by exposing the cells to hypoxia. The ability of ASCs to stimulate the growth of nerve sprouts in ischemic myocardium has recently been demonstrated [6]. Furthermore, nerve conduits seeded with ASCs differentiated towards Schwann-like cell phenotype and promote peripheral nerve repair. However, mechanisms of ASC's action on nerve regeneration are only partially understood. This can be addressed using in vivo models of nerve injury and growth in conjunction with determining gene expression patterns in the cells.

In this study we tested the hypothesis that ASCs stimulate repair of crushed peripheral nerves and induce nerve sprout growth by producing neurotrophic growth factors as well as myelin sheath components. Since the ability of ASCs to prevent hypoxia-induced brain injury is dependent on BDNF production [7], we also examined the impact of this neurotrophin on nerve fiber growth induced by ASCs.

## Materials and Methods

### Ethics statement

Written consent has been given from the patients for their information to be stored in the hospital database and used for research. The use of tissue was approved by the corresponding Ethics Committees of the Faculty of Fundamental Medicine and Cardiology Research Center. Freshly excised clinical specimens included in this study were collected from consenting patients undergoing surgical treatment at Cardiology Research Center.

Animal studies were conducted according to Institute of Experimental Cardiology approved guidelines, as well as guidelines approved by the Institutional Animal Care and Use committee of Cardiology Research Center (permit number 385.06.2009).

### Animals

We used 8–9 week old C57Bl6-GFP transgenic and C57Bl6 male mice as a source of mouse ASCs (mASCs) and also for the matrigel transplantation experiments. F1 offspring of CBA/C57Bl6 mice were used for the nerve injury model. Animals were anesthetized by i.p. injection of avertin before experimental manipulations.

### Cell Isolation, Culture and Stimulation

Human (h) ASCs were isolated from adipose tissue obtained from 15 female donors during abdominal surgery [8]. All donors gave their informed consent and the local ethics committee approved the study protocol. Mouse subcutaneous adipose tissue was obtained from the inguinal region and cells isolated by enzyme digest. Cells were cultured in AdvanceSTEM Mesenchymal Stem Cell Media containing 10% AdvanceSTEM Supplement (HyClone), 1% antibiotic–antimycotic (HyClone) at 37°C in 5% CO2 incubator. Cells were passaged to 70% confluency using HyQT ase solution (HyClone). For the experiments, cells of the second passage were used.

Recently we and others showed that hypoxia up-regulates expression of growth factors by ASCs [9]. Hypoxia was induced by culturing serum-deprived cells in a hypoxic incubator (48R, New Brunswick) at 37°C, using 5% CO_2_ and 1% O_2_ for 48 hours. Cell cultured in hypoxia conditions are identified as hypASCs for mouse ASCs and hyp_hASCs for human ASCs. Induction of neural differentiation was performed using retinoic acid, a well known inducer of neural differentiation [10]. mASCs or hASCs at 40–60% confluence were placed in neural differentiation medium (AdvanceSTEM Mesenchymal Stem Cell Media containing 3% AdvanceSTEM Supplement (HyClone), 1% antibiotic–antimycotic (HyClone), 1 µM 5- azacytidine and 1 µM retinoic acid) for 72 hours. Cells stimulated towards neural differentiation are referred to as indASCs for mouse ASCs and ind_hASCs for human ASCs.

### Nerve injury model

The common peroneal nerve (*n. peroneus communis*) was isolated from surrounding tissue under sterile conditions and crushed using 1-mm-wide needle holder with silicone coated branches 3 times for 20 seconds [11]–[13]. mASCs suspended in matrigel matrix (70 µl) were applied over injured nerve. Mice were divided into three experimental groups: a negative control group, which received matrigel without cells (n = 14); a mASC treatment group, which received matrigel with 1×106 of mASCs (n = 14); and a positive control group, which received matrigel without cells but administered 65 µg/kg vitamin B12 i.p. every day after surgery (n = 14) [14]. After matrigel polymerization the wound was closed. Then the effect of mASC implantation on functional nerve recovery was assessed. Motor nerve recovery was analyzed 1, 2, 4, 7 and 11 days after surgery using the peroneal function index (PFI) [15], [16]. Mouse hind paws were stained with ink and animals were allowed to run along a tunnel, 6×50 cm. Footprint images were scanned and footprint width and length measured using Metamorph 7.1 software. PFI was calculated using the following formula: PFI = 174.9*(EPL−NPL)/NPL+80.3*(ETS−NTS)/NTS−13.4; where EPL and ETS are footprint length and width, respectively, measured 1, 2, 4, 7 or 11 days after injury; and NPL and NTS are footprint length and width, respectively, measured before injury [17]. Sensory nerve recovery was analyzed 7 and 11 days after surgery using electrophysiological parameters on the superficial branch of common peroneal nerve (*n. peroneus superficialis*). Mice were sacrificed with anesthetic overdose (7 animals from each group after performing walking track analysis 7 and 11 days after injury). The superficial branch of common peroneal nerve was isolated and stimulated in Laily solution 7–8 mm proximal from injury site (frequency 1 Hz, impulse length 0.05 ms and supramaximal stimulus amplitude 2.5–10 V) using a pair of silver electrodes, (distance between electrodes 1.5 mm). The waiting period in-between scarification of the animals and measurement of nerve conduction velocity was standard (35–45 minutes) for all the animals. The length of latency period and amplitude of total nerve action potential were recorded 3 times for each nerve using a monopolar aspirating electrode (10 mm distal from injury site) connected to AD-converter and output analyzed using PowerGraph Professional 3.3 software (Figure S1).

The effect of mASCs on nerve healing was then confirmed. Myelin sheaths of healing nerve were analysed using Sudan black staining as described elsewhere [18]. Briefly, frozen sections of peripheral nerve were fixed by 0.4% potassium dichromate, pH 3.5 for 18 hrs at 56°C. Then sections were stained by 0.1% Sudan Black B (Merck) in 70% ethanol for 5 min at room temperature, washed by 50% ethanol, dehydrated and mounted in Depex medium (Merck). The number of axons was evaluated on formalin-fixed frozen sections using immunofluorescent staining with rabbit anti-neurofilament 200 antibody (Abcam, 8135) (see *Immunofluorescent staining*… section below for details). Alexa 488-conjugated anti-rabbit antibody (Molecular Probes) was the secondary antibody. Nuclei were counterstained with 4-, 6-diamidino-2-phenylindole (DAPI) (Molecular Probes). Rabbit non-specific IgG was used as a negative control. Images were obtained using Leica6000 microscope equipped with CCD camera (Leica Microsystems).

### Matrigel Implantation into Mice

The effect of ASCs on axon growth in vivo was assessed using matrigel subcutaneous implant. This method was widely used for assessment of blood vessels growth [4] [19]. Since blood vessels and nerves grow simultaneously, we used this model for studying axon growth. Mice were anesthetized by i.p. injection of 2.5% avertin solution. We mixed 400 µl of ice-cold growth factor reduced matrigel matrix (BD Biosciences) with 7×10^5^ mASCs in 50 µl of AdvanceSTEM Mesenchymal Stem Cell Media (HyClone) and injected obtained suspension subcutaneously into the right and left sides of the mouse's back using insulin syringe with a 23G needle. Injections were performed slowly allowing matrigel to polymerize and form a jelly-like implant with an irregular shape under the skin. We used four different matrigel preparations: one with growth media only (no cells; negative control; n = 9); one containing mASCs cultured in normoxic conditions (norm_mASCs; n = 9); another with mASCs cultured in hypoxic conditions (hyp_mASCs; n = 9) and one containing mASCs, which have been cultured in neural differentiation medium prior to incorporation into matrigel (ind_mASCs; n = 9). These were randomized so that each mouse was implanted with two different matrigel preparations (e.g. one from the negative control preparation and one containing normoxic mASCs). A total of 18 mice received the matrigel implants. Ten days after injection, the mice were killed with an anesthetic overdose and perfused with formalin. Entire matrigel plugs were then isolated, weighted, embedded in O.C.T. compound (Sakura Inc) and frozen in liquid N_2_. To determine whether BDNF contributes to the effects of mASCs on nerve fiber growth, we also implanted into 5 mice matrigel containing mASCs plus non-immune IgG (10 µg/ml) and matrigel containing mASCs plus BDNF neutralizing antibodies (10 µg/ml), one implant of each per mouse, which were examined 10 days later.

### Immunofluorescent staining of matrigel sections and analysis of nerve fiber density

Nerve fibers in matrigel were detected by immunofluorescent staining using antibodies to nerve marker proteins GAP43 (Abcam, cat # ab12274), neurofilaments 200 (Sigma, cat # 4142), peripherin (Abcam, cat # 4666) and tyrosine hydroxylase (Abcam, cat # 112). Alexa 594-conjugated anti-rabbit antibody (Molecular Probes) was the secondary antibody. Nuclei were counterstained with 4-, 6-diamidino-2-phenylindole (DAPI) (Molecular Probes). For negative controls rabbit non-specific IgGs were used. Images were obtained using Leica6000 microscope equipped with CCD camera (Leica Microsystems). Quantitation of nerve fiber density and length was performed on 6 frozen sections from each matrigel implant using immunofluorescence of the nerve growth marker protein GAP43 [20], [21]. Nerve length and density were evaluated using MetaMorph 7.1 (Molecular Devices) as described earlier [22].

### RNA extraction from ASC, RT-PCR and real-time PCR Analysis

To determine how culture of ASCs in hypoxic conditions and differentiation media affects expression of neurotrophins, total cellular RNA was isolated from mASCs or hASCs using RNeasy Kit (Qiagen, cat # 74104) following by a reverse transcription reaction using a Reverse Transcription kit (Fermentas, cat # K1611). Real-time polymerase chain reactions (PCR) were performed using a SYBR Green PCR Master Mix (Fermentas, cat # K0221) and the Multicolor Q5 (Bio-Rad). PCR reactions were performed using 50 ng of cDNA and 100–500 nmol/L of each primer. For all reaction the thermal cycling parameters were: 10 minutes at 95°C followed by 40 cycles of 15 seconds at 95°C for denaturation, 20 seconds at empirically adjusted temperature for each primer to anneal and 20 seconds at 72°C to extend. We used following oligonucleotide primers for PCR analysis of gene expression in hASCs: beta-actin for CCTGGCACCCAGCACAAT, beta-actin rev GGGCCGGACTCGTCATAC; GAPDH for TGCACCACCAACTGCTTAGC, GAPDH rev GGCATGGACTGTGGTCATGAG; VEGF for CAACATCACCATGCAGATTATGC (60.2°C), VEGF rev CCCACAGGGATTTTCTTGTCTT (60.2°C); BDNF2 for GTAGTCTTCTTGGCCCCGCTGTAA (59.6°C), BDNF2 rev AACGCTCCGCTCCAAAATCTGA (59.7°C); GDNF1 for GCTGTCTGCCTGGTGCTGCTC (59.6°C), GDNF1 rev GCCTGCCGATTCCGCTCTC (59.3°C); NGF for AGGGAGCAGCTTTCTATCCTG (61°C), NGF rev GGCAGTGTCAAGGGAATGC (61.1°C). We used following oligonucleotide primers for PCR analysis of gene expression in mASCs: beta-actin for AGTGTGACGTTGACATCCGTA, beta-actin rev GCCAGAGCAGTAATCTCCTTCT; GAPDH for GACCCCTTCATTGACCTCAACTAC, GAPDH rev TGGTGGTGCAGGATGCATTGCTGA; VEGF for GCAAAACACTCACCATTCCCA (60.2°C), VEGF rev GAGGTTTGAAATCGACCCTCG (60°C); BDNF2 for TTTGCGGCATCCAGGTAATTT (59.6°C), BDNF2 rev CCATAAGGACGCGGACTTGTA (59.7°C); GDNF1 for CCAGTGACTCCAATATGCCTG (60°C), GDNF1 rev CTCTGCGACCTTTCCCTCTG (59°C); NGF for GCACTACACCCATCAAGTTCA (61.5°C), NGF rev TCCTGAGTCATGCTCACAAGT (61.1°C). All measurements were performed in triplicates. Levels of expression were normalized to the expression of 2 house-keeping genes GAPDH and beta-actin.

### Transcriptome analysis

To determine whether ASCs express neurotrophins and matrix components required for axonal growth, we performed gene array experiments using cultured hASCs. Five hundred nanograms of total RNA was labeled and hybridized on HumanHT-12 v4 Expression BeadChip (Cat. no. BD-103-0204; Illumina, San Diego, CA, USA), according to the manufacturers recommendations (Illumina Gene Expression Profiling Assay Guide). BeadChips were scanned with the Illumina iScan Reader. Data were imported into GenomeStudio (Illumina) and analyzed.

### Measurement of BDNF Secretion by ELISA

hASCs were cultured in serum-free medium under hypoxic conditions, or in the presence of 5-azacitidin or 5-azacitidin plus retinoic acid for 2 days, then medium was collected and the concentration of BDNF in the media determined using a BDNF ELISA kit (Millipore, cat #GYT306), following the manufacturer instructions.

### Statistical Analysis

Data was assessed for normality of distribution using the Kolmogorov-Smirnov test. Statistical analysis was performed using SigmaPlot11.0 Software. Differences between treatment and control groups were then analyzed using Student t-test or the Mann-Whitney U-test, depending whether it was normally distributed or not. Data are expressed as mean ± SEM or median (25%; 75%) depending on the test used. We considered differences to be significant when p<0.05.

## Results

ASCs can induce tissue regeneration and were suggested as potent candidates for cell therapy. To assess their action on nerve growth and repair we have employed in vivo models accompanied by expression analysis of genes involved in the regulation of axonal growth and myelination.

### mASCs stimulate repair of injured nerves

**Figure 1:**
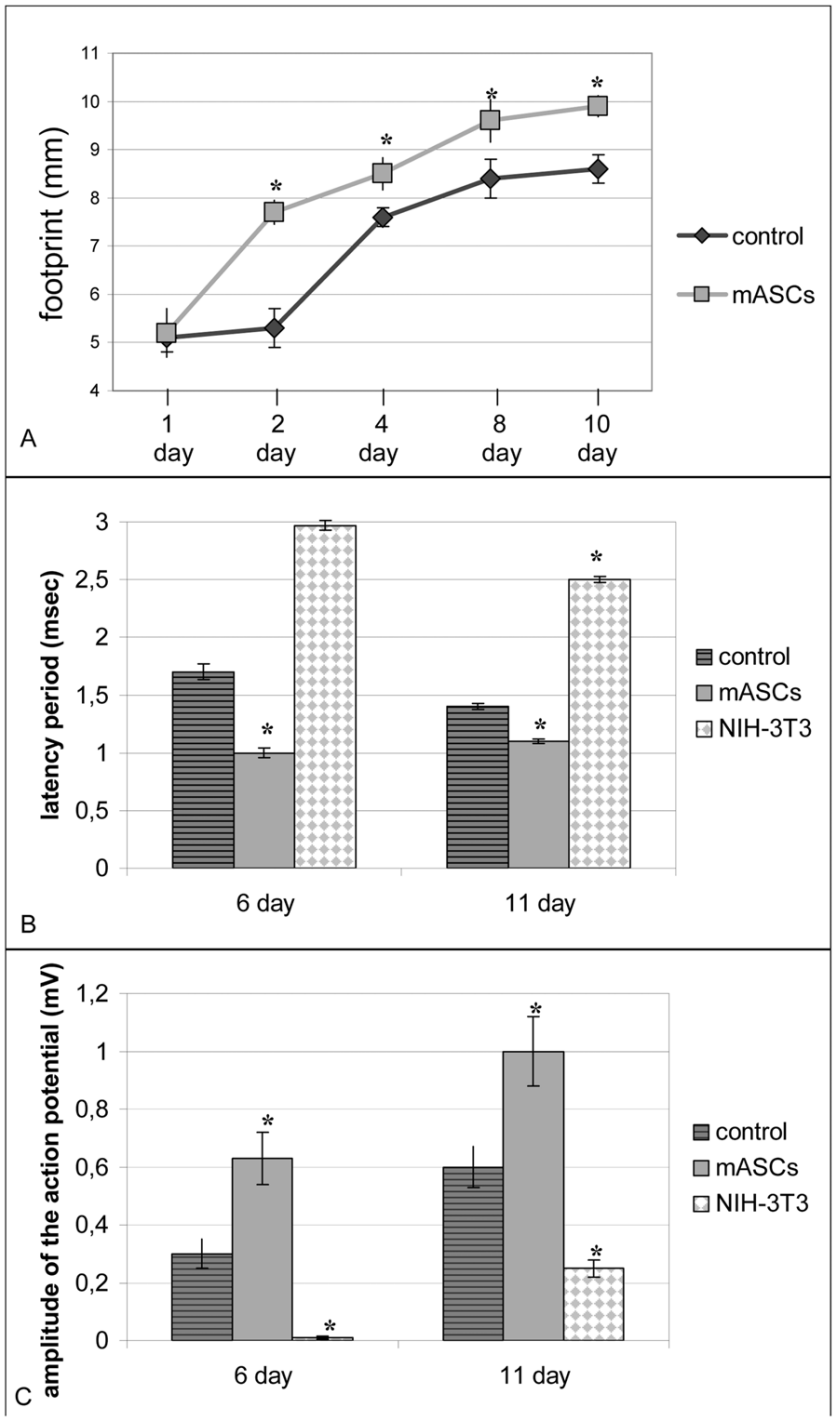
Recovery of common peroneal nerve functioning after mASCs transplantation. A.– peroneal function index (PFI) recovery (motor nerve functioning). B, C. – recovery of nerve conduction velocity (sensory nerve functioning): B. – action potential amplitude; C. - latency period. * - p<0.05.

Application of mASCs to the injury site of common peroneal nerve accelerated functional recovery of motor and sensory nerves. The extensor muscles of the toes exhibited improved function in animals treated with mASCs comparing to negative controls, reflecting improved activity of respective motor neurons. As early as 2 days after injury, peroneal functional index (PFI) [15] of the injured limbs of animals treated with mASCs was greater comparing to negative controls (−80.4 (−88.2; −74.7), n = 84 vs. −110.5 (−115.8; −106.0), n = 84; *p*<0.001), and also positive controls (−109.8 (−121.7; −106.5), n = 84; *p*<0.001), indicating faster recovery of motor nerves exposed to an ASCs (Figure 1A). The PFI of injured limbs of animals treated with mASCs was also greater compared to negative control group and positive control group 4 days after injury (−38.5 (−67.1; −28.5), n = 84 vs. −74.9 (−80.1; −57.3), n = 84, *p*≤0.001; and −82.8(−87.3; −73.4), n = 84, *p*≤0.001, respectively). Despite PFI of mice treated with mASCs was greater compared to negative control group 7 days after injury (−42.8 (−48.0; −16.1), n = 84, vs. −65.2 (−66.7; −64.5), n = 84, *p*≤0.001) it did not differ from positive control group (−44.0 (−54.1; −37.9), n = 84, *p* = 0.075).

We also observed significant recovery of the action potential amplitude together with a reduction in the latency period of sensory neurons of common peroneal nerve in mice treated with mASCs (Figure 1B, C). Action potential amplitude of the superficial branch of common peroneal nerve in animals treated with mASCs 7 days after injury was 3.7-fold and 2-fold greater compared to negative control group and positive control group (0.82 (0.39;0.92), n = 21 vs. 0.22 (0.15; 0.29), n = 21, *p*≤0.001 and 0.42 (0.36; 0.45), n = 21, *p*≤0.001, respectively). This suggests that more nerve fibers were participating in signal conduction. This effect was sustained 11 days after injury. The latency period of the nerve treated with mASCs 7 days after injury was 1.7-fold and 1.3-fold shorter compared to negative control group and positive control group (1.07 (0.97; 1.11), n = 21 vs. 1.7(1.65; 2.1), n = 21, *p*≤0.001 and 1.30(1.27; 1.4), n = 21, *p*≤0.001 respectively). This indicates better myelination of nerve fibers and greater thickness of nonmyelinated nerve fibers. However, the difference of the latency period of nerve conduction velocity between treated animals and negative control was less prominent 11 days after injury (1.09 (1.07; 1.23), n = 21 vs. 1.46 (1.34; 1.51), n = 21, p<0.05).

Also, we observed more NF200 positive axons with thicker myelin sheaths in nerves treated with mASCs 7 days after injury (Figure S2), which confirm functional data and indicate that mASCs application accelerates nerve healing.

### mASCs stimulate nerve fiber growth in matrigel implants *in vivo*

**Figure 2:**
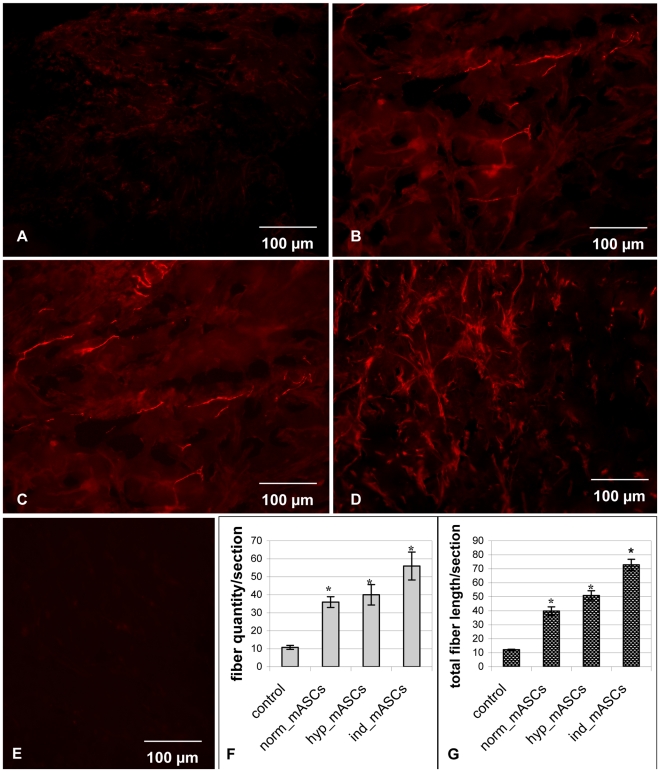
GAP43-positive nerve fibers in matrigel. A–D. - Immunofluorescent staining of frozen sections of matrigels with GAP43 antibodies (red fluorescence). A. – matrigel with growth media only (no cells; negative control). B. – matrigel containing mASCs cultured in normoxic conditions (norm_mASCs). C. - matrigel with mASCs cultured in hypoxic conditions (hyp_mASCs). D. - matrigel containing mASCs which have been cultured in neural differentiation medium prior to incorporation into matrigel (ind_mASCs). E. – frozen matrigel sections stained with non-specific IgGs. F. – density of nerve fibers (pieces) per section. G. – total length (relative units) of nerve fibers. * - p<0.05.

Because mASCs enhanced repair of damaged nerves, we next assessed whether they might stimulate nerve fiber growth. The ability of mASCs to stimulate nerve fiber growth was assessed in matrigel implants. Ten days after transplantation matrigel implants contained rare structures expressing the marker nerve growth cone GAP43 (Figure 2A). The transplantation of mASCs elevated nerve fiber density in matrigel implants. The number of GAP43-positive structures was 3.4-fold greater in implants containing norm_mASCs compared to negative control group (36.27±1.98, n = 54 vs. 10.76±1.27, n = 54, p = 0.008; Figure 2B, F). The total length of observed structures was also 3.1-fold greater in implants containing norm_mASCs compared to negative control group (39.93±2.38, n = 54 vs. 12.91±1.52, n = 54, p<0.001; Figure 2B, G). Nerve fiber length in matrigel implants containing pre-stimulated cells was even greater, 1.3-fold in implants containing hyp_mASCs (50.43±2.03, n = 54, p = 0.045 vs. norm_mASCs; Figure 2C, G) and 1.8-fold in implants containing ind_mASCs (72.62±1.8, n = 54, p = 0.007 vs. norm_mASCs; Figure 2D, G).

**Figure 3:**
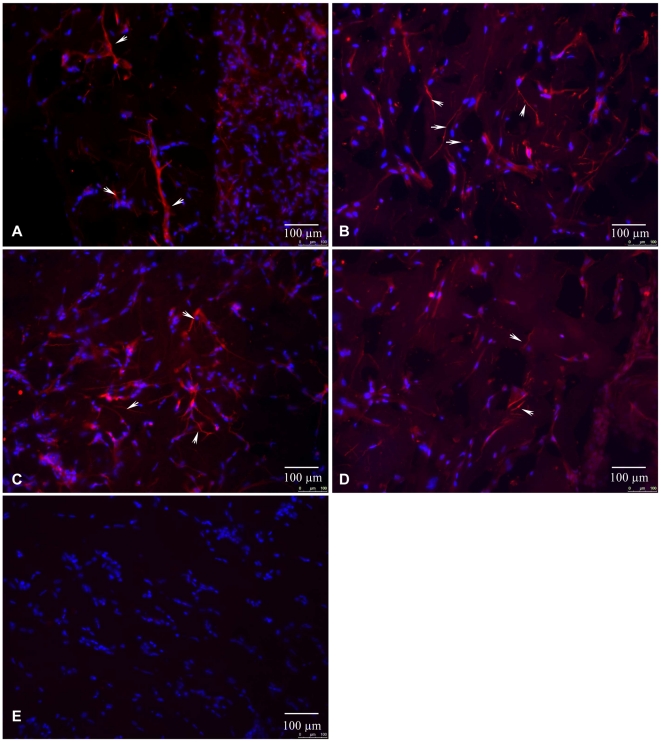
Expression of peripheral nerves markers in matrigel after mASCs transplantation. A–E. - Immunofluorescent staining of frozen matrigel sections with antibodies against neurofilament200 (A.), GAP43 (B.), tyrosine hydroxylase (C.), peripherin (D) or with non-specific IgGs (red fluorescence). Nerve fibers are indicated by arrows. Nuclei are counterstained with DAPI (blue fluorescence).

To confirm that mASCs stimulated the growth of nerve fibers expressing GAP43, we analyzed the expression of additional peripheral nerve markers in the matrigel implants. The matrigel implants contained structures expressing axonal cytoskeleton protein, neurofilament 200, intermediate filament subunit concentrated in processes of peripheral ganglia neurons, peripherin, and tyrosine hydroxylase, an enzyme involved in the biosynthesis of noradrenalin in sympathetic neurons (Figure 3). These structures were derived from peripheral nerves innervating the skin and muscles adjacent to the matrigel implants. During the waiting period (10 days) after subcutaneous implantation of matrigel there was some reduction in the matrigel gel plugs but it was never more than 20% and similar for all the treatment groups (p for differences >0.05). At this time mASC numbers in the matrigel were 10% of those originally implanted and were viable. Also, we found no evidence to support possible in vivo differentiation of the mASCs into neural lineage cells. mASCs from GFP transgenic mice in matrigel implants did not colocalise with GAP43, NF200, S100 or GFAP detected by immunofluorescence (not shown).

### hACSs express neurotrophins and matrix components required for axonal growth

Analysis of 24,500 transcripts revealed that hASCs expressed 10,995 known transcripts (p<0.05). About 200 of these transcripts encoded for specific neurogenesis-related proteins including enzymes, cytoskeleton proteins and adhesion molecules. Importantly, hASCs and mASCs (not shown) expressed mRNAs encoding neurotrophins and neuregulins, including BDNF, NGF, NENF, GDNF and neuregulin 1, suggesting that these cells could be the source of neurotrophins at the site of injury. Furthermore, hASCs expressed neurite guidance molecules such as netrins and ninjurin 2 as well as extracellular matrix proteins such as laminins. Importantly, hASCs expressed myelination master-gene Krox20 (Egr2) together with transcripts encoding components of peripheral myelin, including proteolipid protein 1, peripheral myelin protein 22, periaxin, 3 transcript variances of myelin basic protein as well as myelin proteins zero-like 1 and 2 (for details see Table S1).

**Figure 4:**
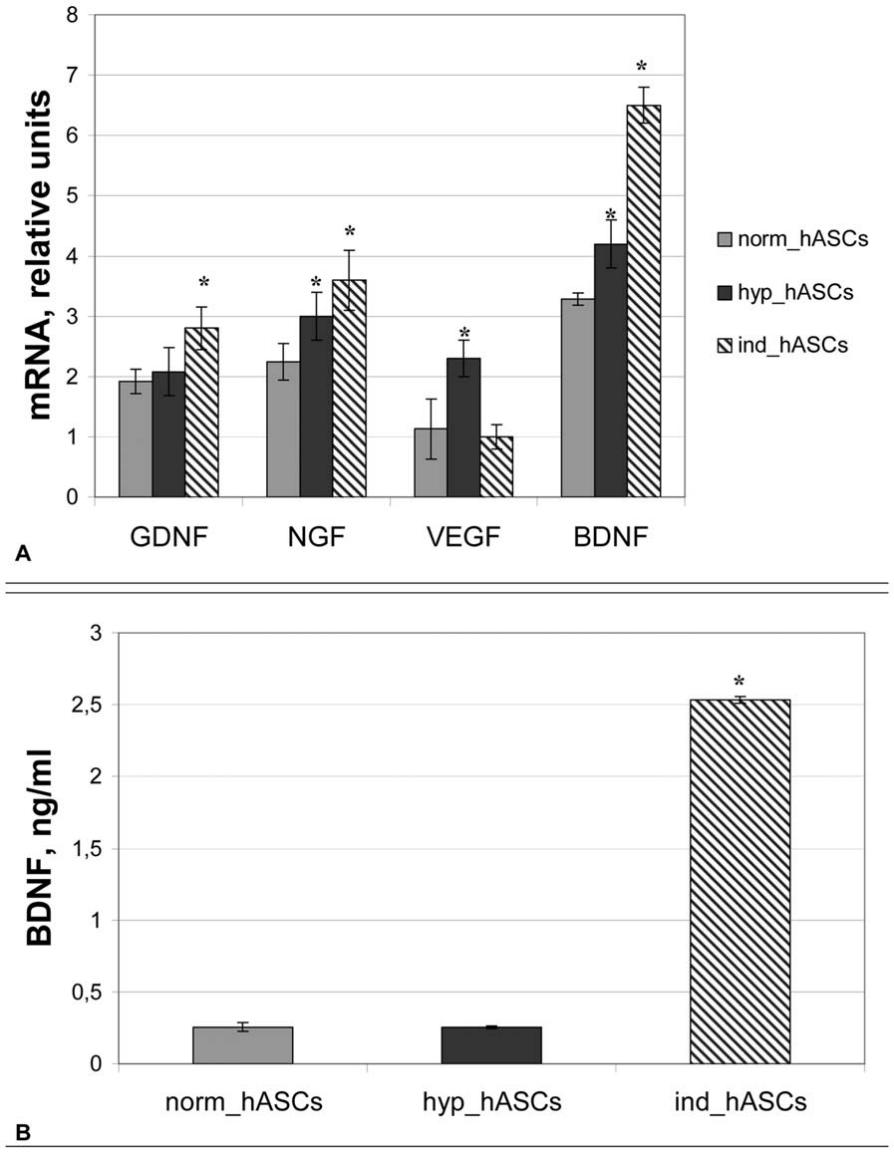
Neurotrophin expressions by hASCs. A.- Real-time PCR analysis of neurotrophin mRNAs expressions in hASCs cultured in standard conditions (norm_hASCs, grey columns), in hypoxia (hyp_hASCs, black columns) or neural differentiation medium (ind_hASCs, hatched columns). B. – ELISA of BDNF level in conditioned medium of hASCs cultured in standard conditions (grey columns), in hypoxia (black columns) or neural differentiation medium (hatched columns). *-p<0,05 compared to control.

Hypoxia and culturing in neural differentiation induction medium up-regulated the expression of neurotrophins in hASCs. Hypoxia elevated BDNF mRNA by 50% and NGF by 25%, but did not affect the expression of GDNF (Figure 4A). The incubation of hASCs in neural differentiation induction medium caused even greater up-regulation of neurotrophin expression. In contrast to hypoxia, neural differentiation medium did not elevate the expression of VEGF (Figure 4A). We evaluated how hypoxia and neurogenic induction medium affected the secretion of the neurotrophic factor, BDNF. Surprisingly, hypoxia did not affect BDNF secretion by hASCs. In contrast, culture medium from hASCs treated with 5- azacytidin plus retinoic acid contained up to 10-fold more BDNF compared to control (22±5 ng/ml, n = 9 vs. 3±1.2 ng/ml, n = 9; p<0.001).

### BDNF takes part in the stimulation of nerve fiber growth

**Figure 5:**
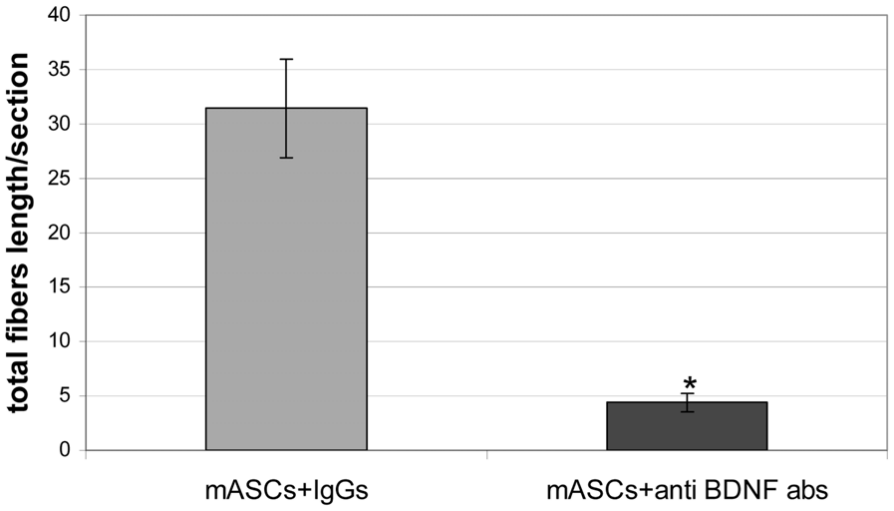
Total length of GAP43-positive nerve fibers in matrigel. Total length of nerve fibers per section was analyzed 10 days after transplantation using immunofluorescent staining with GAP43 antibodies of frozen sections of matrigel containing mASCs with 10 µg/ml non-specific IgGs (hatched column) or anti-BDNF neutralizing antibodies (grey column). * - p<0,05.

To determine if the observed stimulation of nerve fiber growth was dependent on BDNF production by ASCs, mASCs were suspended in matrigel together with 10 µg/ml of a rabbit polyclonal BDNF neutralizing antibody or non-specific rabbit IgG and then implanted into mice. 10 days later we assessed the density of nerve fibers in the implants. Total length of nerve fibers in the matrigel implants containing mASCs and BDNF neutralizing antibodies was about 9 times smaller compared to implants with cells mixed with non-specific IgGs (3.4±0.9, n = 30 vs. 30.7±5.2, n = 30; p<0.001) (Figure 5).

## Discussion

Our study demonstrates that transplantation of mASCs induces peripheral nerve repair and activates nerve sprout growth in vivo. Also examination of gene expression profile of mASCs and hASCs indicated that these cells produce neurotrophic factors and myelin sheath components, which are necessary for nerve sprout outgrowth and myelination. Our study demonstrates that ability of mASCs to stimulate the growth of nerve sprouts depends on BDNF secretion.

Transplantation of mASCs induces the growth of blood vessels and nerve sprouts in ischemic myocardium, stimulating regeneration [6]. ASCs differentiated towards Schwann-like cell phenotype also promote neurite outgrowth [23]. Furthermore, nerve conduits seeded with such cells enhance myelination [24] and repair of peripheral nerve [6]. Here we demonstrate that mASCs affect the growth of tyrosine hydroxylase expressing sympathetic nerve sprouts. Furthermore, mASCs transplantation stimulated a functional recovery of crushed sensory and motor neurons, indicating that this effect is common for all nerve fibers rather than selective for particular type of neurons. In addition, nerves treated with mASCs exhibited faster structural recovery. One explanation for those effects is their ability to produce growth factors, such as VEGF, bFGF and HGF together with neurotrophins, BDNF, NGF, GDNF and NT-1 [7], [25], [26]. Neurotrophins stimulate nerve fiber growth and regeneration in several ways: they directly accelerate axonal growth via PI-3K or PLC-γ - dependent signaling pathways [27], [28]; act as positive guidance molecules for axonal growth cone, prevent apoptosis and induce proliferation of Schwann cells. Furthermore, ASCs can elevate neurotrophins production indirectly at the injury site by stimulating the growth of new blood vessels. Vascular cells produce BDNF and artemin (ARTN), a neurotrophin of GDNF family, which attract growing sympathetic nerve fiber and simultaneously promote nerve fiber formation along the blood vessel [29]. Neurotrophic activity of vascular cells attracted to the injury site by transplanted ASCs could be responsible for a long-term effect of those cells.

We demonstrate that hypoxia further increases the ability of ASCs to stimulate the growth of nerve fibers. This reconciles our previous observations that low-oxygen conditions up-regulate their angiogenic capabilities and supports the idea that ASCs transplantation induces the simultaneous growth of nerve sprouts and blood vessels. Indeed, we found that in matrigel implants GAP43 positive nerve fibers which co-localized with lectin-positive blood vessels (Lopatina T, unpublished). Neural differentiation medium also increased the ability of ASCs to stimulate nerve fiber growth. Hypoxia and neural differentiation enhance the paracrine activity of ASCs [4], [9], [25], [30]. We show that low oxygen up-regulates angiogenic growth factors with modest effects on neurotrophins, and the combination of retinoic acid with 5-azacytidin further stimulates expression of neurotrophins and their secretion without influencing VEGF. Among the neurotrophins produced by ASCs, BDNF is likely to play the important role. However, we observed changes in BDNF secretion only in ASCs incubated in differentiation medium, whereas when the cells were cultured under hypoxic conditions we found only an increase of BDNF mRNA. This observation might be explained by the fact that production of BDNF protein is regulated at the level of translation. Thus, the long BDNF 3′UTR has been reported as a bona fide cis-acting translation suppressor of BDNF mRNA [31]. Furthermore, BDNF translation is also regulated by microRNA, e.g. miR-30a-5p targets specific sequences surrounding the proximal polyadenylation site within BDNF 3′-untranslated region and overexpression of this miR results in down-regulation of BDNF protein [32]. Thus, prevention of hypoxia-induced brain damage by conditioned medium from ASCs has been attributed to BDNF secretion [7]. Furthermore, adenoviruses encoding BDNF have been used to stimulate axonal regeneration [33]. We demonstrate that the ability of ASCs to up-regulate nerve sprouts growth correlates with their production of BDNF and that anti-BDNF neutralizing antibodies abrogated their stimulatory effect, indicating that BDNF is an important mediator of ASCs actions. Growing nerve sprouts migrate along specific matrix components, including laminins [34]. Just like bone marrow-derived MSCs, ASCs also express several genes of the laminin family, indicating that these cells can directly support growing nerve sprouts [35]. Thus, ASCs play a role analogous to Schwann cells at sites of transplantation. Interestingly, transcriptome analysis revealed the expression of neural marker genes by ASCs, including nestin, beta3-tubulin and neurofilament 150. Furthermore, these cells appeared to express myelination master-gene Krox20 [36] and its transcriptional targets, major components of myelin sheath [37]. While this manuscript was in preparation a study was published showing that MSCs derived from bone marrow and adipose tissue express mRNAs encoding several myelin components and co-culture with neural cells stimulates the secretion of these proteins [38]. Our functional and histological data suggest that faster healing of crushed common peroneal nerve is due at least in part to restoration or protection of the myelin sheath by mASCs application. Either contact with injured nerves or neural differentiation medium triggers the myelination program by ASCs. The reason as to why ASCs exhibit an expression profile similar to Schwann cells might be due to their similar embryonic origin [39]. This clearly requires further study.

Taken together, our data suggest that ASCs similar to Schwann cells can provide neurotrophic growth factors to injured nerves and improving their re-myelination. Incubation in hypoxic conditions or in neural differentiation medium prior to transplantation increases their regenerative potential, which depends on the production of neurotrophins, particularly BDNF. Therefore, ASCs might be a useful cell therapy for regeneration of injured peripheral nerves and their re-myelination.

## Supporting Information

Figure S1 **Design of nerve injury model.** A – photography of n. peroneus communis separated from surrounding tissue before injury. B - photography of n. peroneus communis injury site. C. - scheme of nerve conduction velocity assessment.
(TIF)

Figure S2 **Common peroneal nerve healing 7 days after injury**. A–D. - Immunofluorescent staining of frozen sections with NF-200 antibody (green fluorescence). E–H. - Staining of frozen sections with Sudan black (blue color). A, E. – uninjured nerve. B, F. – injured nerve treated with matrigel only (no cells; negative control). C, G. – injured nerve treated with mASCs. D, H. - injured nerve with matrigel only but administered 65 µg/kg vitamin B12 i.p. (positive control).
(TIF)

Table S1 (DOC)

## References

[1] Walsh S, Midha R (2009). Use of stem cells to augment nerve injury repair.. Neurosurgery.

[2] Fuhrmann T, Montzka K, Hillen LM, Hodde D, Dreier A (2010). Axon growth-promoting properties of human bone marrow mesenchymal stromal cells.. Neurosci Lett.

[3] Guest JD, Rao A, Olson L, Bunge MB, Bunge RP (1997). The ability of human Schwann cell grafts to promote regeneration in the transected nude rat spinal cord.. Exp Neurol.

[4] Rubina K, Kalinina N, Efimenko A, Lopatina T, Melikhova V (2009). Adipose stromal cells stimulate angiogenesis via promoting progenitor cell differentiation, secretion of angiogenic factors, and enhancing vessel maturation.. Tissue Eng Part A.

[5] Traktuev DO, Parfenova EV, Tkachuk VA, March KL (2006). [Adipose stromal cells–plastic type of cells with high therapeutic potential].. Tsitologiia.

[6] Cai L, Johnstone BH, Cook TG, Tan J, Fishbein MC (2009). IFATS collection: Human adipose tissue-derived stem cells induce angiogenesis and nerve sprouting following myocardial infarction, in conjunction with potent preservation of cardiac function.. Stem Cells.

[7] Wei X, Du Z, Zhao L, Feng D, Wei G (2009). IFATS collection: The conditioned media of adipose stromal cells protect against hypoxia-ischemia-induced brain damage in neonatal rats.. Stem Cells.

[8] Zuk PA, Zhu M, Mizuno H, Huang J, Futrell JW (2001). Multilineage cells from human adipose tissue: implications for cell-based therapies.. Tissue Eng.

[9] Efimenko A, Starostina EE, Rubina KA, Kalinina NI, Parfenova EV (2010). [Viability and angiogenic activity of mesenchymal stromal cells from adipose tissue and bone marrow in hypoxia and inflammation in vitro].. Tsitologiia.

[10] Zhang XM, Li QM, Su DJ, Wang N, Shan ZY (2009). RA induces the neural-like cells generated from epigenetic modified NIH/3T3 cells.. Mol Biol Rep.

[11] Balezina OP, Gerasimenko N, Dugina TN, Strukova SM (2004). [Characteristic properties of thrombin neurotropic activity].. Usp Fiziol Nauk.

[12] Balezina OP, Gerasimenko NY, Dugina TN, Strukova SM (2005). Study of neurotrophic activity of thrombin on the model of regenerating mouse nerve.. Bull Exp Biol Med.

[13] Vard'ya IV, Mospanova SV, Portnov VV, Balezina OP, Koshelev VB (2000). Interval training by normobaric hypoxia accelerates the reinnervation of musculus extensor digitorum longus in mice.. Dokl Biol Sci.

[14] Yuan Y, Shen H, Yao J, Hu N, Ding F (2010). The protective effects of Achyranthes bidentata polypeptides in an experimental model of mouse sciatic nerve crush injury.. Brain Res Bull.

[15] Bain JR, Mackinnon SE, Hunter DA (1989). Functional evaluation of complete sciatic, peroneal, and posterior tibial nerve lesions in the rat.. Plast Reconstr Surg.

[16] Yuan Q, Hu B, Su H, So KF, Lin Z (2009). GAP-43 expression correlates with spinal motoneuron regeneration following root avulsion.. J Brachial Plex Peripher Nerve Inj.

[17] Kah-Hui Wong MN, Pamela David, Mahmood Ameen Abdulla, Noorlidah Abdullah, Umah Rani Kuppusamy, Vikineswary Sabaratnam (2010). Peripheral Nerve Regeneration Following Crush Injury to Rat Peroneal Nerve by Aqueous Extract of Medicinal Mushroom Hericium erinaceus (Bull.: Fr) Pers. (Aphyllophoromycetideae)..

[18] Plant GW, Currier PF, Cuervo EP, Bates ML, Pressman Y (2002). Purified adult ensheathing glia fail to myelinate axons under culture conditions that enable Schwann cells to form myelin.. J Neurosci.

[19] Passaniti A, Taylor RM, Pili R, Guo Y, Long PV (1992). A simple, quantitative method for assessing angiogenesis and antiangiogenic agents using reconstituted basement membrane, heparin, and fibroblast growth factor.. Lab Invest.

[20] McDonald DM, Bowden JJ, Baluk P, Bunnett NW (1996). Neurogenic inflammation. A model for studying efferent actions of sensory nerves.. Adv Exp Med Biol.

[21] Curtis R, Stewart HJ, Hall SM, Wilkin GP, Mirsky R (1992). GAP-43 is expressed by nonmyelin-forming Schwann cells of the peripheral nervous system.. J Cell Biol.

[22] Rubina K, Kalinina N, Potekhina A, Efimenko A, Semina E (2007). T-cadherin suppresses angiogenesis in vivo by inhibiting migration of endothelial cells.. Angiogenesis.

[23] Kingham PJ, Kalbermatten DF, Mahay D, Armstrong SJ, Wiberg M (2007). Adipose-derived stem cells differentiate into a Schwann cell phenotype and promote neurite outgrowth in vitro.. Exp Neurol.

[24] Xu Y, Liu L, Li Y, Zhou C, Xiong F (2008). Myelin-forming ability of Schwann cell-like cells induced from rat adipose-derived stem cells in vitro.. Brain Res.

[25] Rehman J, Traktuev D, Li J, Merfeld-Clauss S, Temm-Grove CJ (2004). Secretion of angiogenic and antiapoptotic factors by human adipose stromal cells.. Circulation.

[26] Wei X, Zhao L, Zhong J, Gu H, Feng D (2009). Adipose stromal cells-secreted neuroprotective media against neuronal apoptosis.. Neurosci Lett.

[27] Batistatou A, Greene LA (1991). Aurintricarboxylic acid rescues PC12 cells and sympathetic neurons from cell death caused by nerve growth factor deprivation: correlation with suppression of endonuclease activity.. J Cell Biol.

[28] Inagaki N, Thoenen H, Lindholm D (1995). TrkA tyrosine residues involved in NGF-induced neurite outgrowth of PC12 cells.. Eur J Neurosci.

[29] Honma Y, Araki T, Gianino S, Bruce A, Heuckeroth R (2002). Artemin is a vascular-derived neurotropic factor for developing sympathetic neurons.. Neuron.

[30] Lee EY, Xia Y, Kim WS, Kim MH, Kim TH (2009). Hypoxia-enhanced wound-healing function of adipose-derived stem cells: increase in stem cell proliferation and up-regulation of VEGF and bFGF.. Wound Repair Regen.

[31] Lau AG, Irier HA, Gu J, Tian D, Ku L (2010). Distinct 3′UTRs differentially regulate activity-dependent translation of brain-derived neurotrophic factor (BDNF).. Proc Natl Acad Sci U S A.

[32] Mellios N, Huang HS, Grigorenko A, Rogaev E, Akbarian S (2008). A set of differentially expressed miRNAs, including miR-30a-5p, act as post-transcriptional inhibitors of BDNF in prefrontal cortex.. Hum Mol Genet.

[33] Koda M, Kamada T, Hashimoto M, Murakami M, Shirasawa H (2007). Adenovirus vector-mediated ex vivo gene transfer of brain-derived neurotrophic factor to bone marrow stromal cells promotes axonal regeneration after transplantation in completely transected adult rat spinal cord.. Eur Spine J.

[34] Yu WM, Yu H, Chen ZL (2007). Laminins in peripheral nerve development and muscular dystrophy.. Mol Neurobiol.

[35] Fuhrmann T, Montzka K, Hillen LM, Hodde D, Dreier A (2010). Axon growth-promoting properties of human bone marrow mesenchymal stromal cells.. Neurosci Lett.

[36] Decker L, Desmarquet-Trin-Dinh C, Taillebourg E, Ghislain J, Vallat JM (2006). Peripheral myelin maintenance is a dynamic process requiring constant Krox20 expression.. J Neurosci.

[37] Jahn O, Tenzer S, Werner HB (2009). Myelin proteomics: molecular anatomy of an insulating sheath.. Mol Neurobiol.

[38] Mantovani C, Mahay D, Kingham M, Terenghi G, Shawcross SG (2010). Bone marrow- and adipose-derived stem cells show expression of myelin mRNAs and proteins.. Regen Med.

[39] Billon N, Monteiro MC, Dani C (2008). Developmental origin of adipocytes: new insights into a pending question.. Biol Cell.

