# Correction: Adipose-Derived Stem Cells Stimulate Regeneration of Peripheral Nerves: BDNF Secreted by These Cells Promotes Nerve Healing and Axon Growth *De Novo*

**DOI:** 10.1371/journal.pone.0219946

**Published:** 2019-07-12

**Authors:** Tatiana Lopatina, Natalia Kalinina, Maxim Karagyaur, Dmitry Stambolsky, Kseniya Rubina, Alexander Revischin, Galina Pavlova, Yelena Parfyonova, Vsevolod Tkachuk

Following publication of this article [1], concerns were raised regarding a region of similarity between the immunofluorescence images in Fig 2B and 2C that are reported to show nerve fibers in matrigel implants containing mASCs cultured under differing conditions. The image used in Fig 2B is incorrect.

Here the authors provide a revised Fig 2 using alternative images representing the experimental conditions in panels B and C. These alternative images were acquired during the same original experiment. The nerve fiber density and length data reported in Fig 2F and 2G were recorded from counts of 6 frozen sections from each Matrigel implant; after the data were collected, representative images were selected that reflected the data, and the error in image selection does not affect the data shown in the charts. The revised Fig 2 is included here.

**Fig 2:**
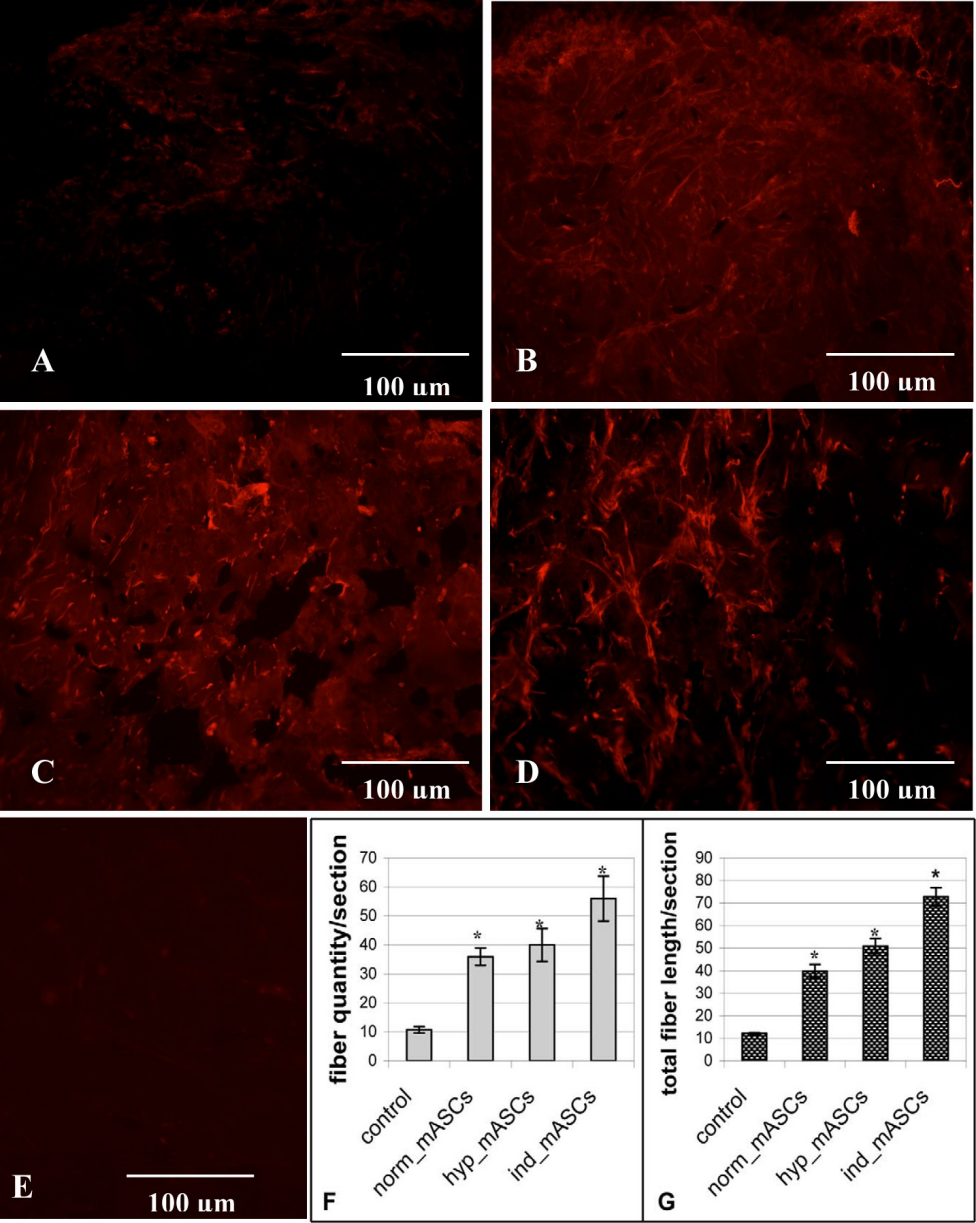
GAP43-positive nerve fibers in matrigel. A–D.—Immunofluorescent staining of frozen sections of matrigels with GAP43 antibodies (red fluorescence). A.–matrigel with growth media only (no cells; negative control). B.–matrigel containing mASCs cultured in normoxic conditions (norm_mASCs). C.—matrigel with mASCs cultured in hypoxic conditions (hyp_mASCs). D.—matrigel containing mASCs which have been cultured in neural differentiation medium prior to incorporation into matrigel (ind_mASCs). E.–frozen matrigel sections stained with non-specific IgGs. F.–density of nerve fibers (pieces) per section. G.–total length (relative units) of nerve fibers. *—p <0.05.

The original images and the nerve fiber density and length data underlying the panels in Fig 2 are not available. The original image files underlying the replacement panels for Fig 2B and 2C are provided as Supporting Information.

S1 Table of the original article reports a gene expression array experiment. The data from the microarray experiment have been deposited at GEO, accession number [GSE130393]. The data underlying the other figures are no longer available.

## Supporting information

S1 File The original image file for replacement Fig 2B. (TIFF)

S2 File The original image file for replacement Fig 2C. (TIFF)
