# Supplementary figures and images for: Adipose-Derived Stem Cells Stimulate Regeneration of Peripheral Nerves: BDNF Secreted by These Cells Promotes Nerve Healing and Axon Growth De Novo
Source: PLoS One. 2011 Mar 14;6(3):e17899. doi: 10.1371/journal.pone.0017899 (PMC3056777; doi:10.1371/journal.pone.0017899)

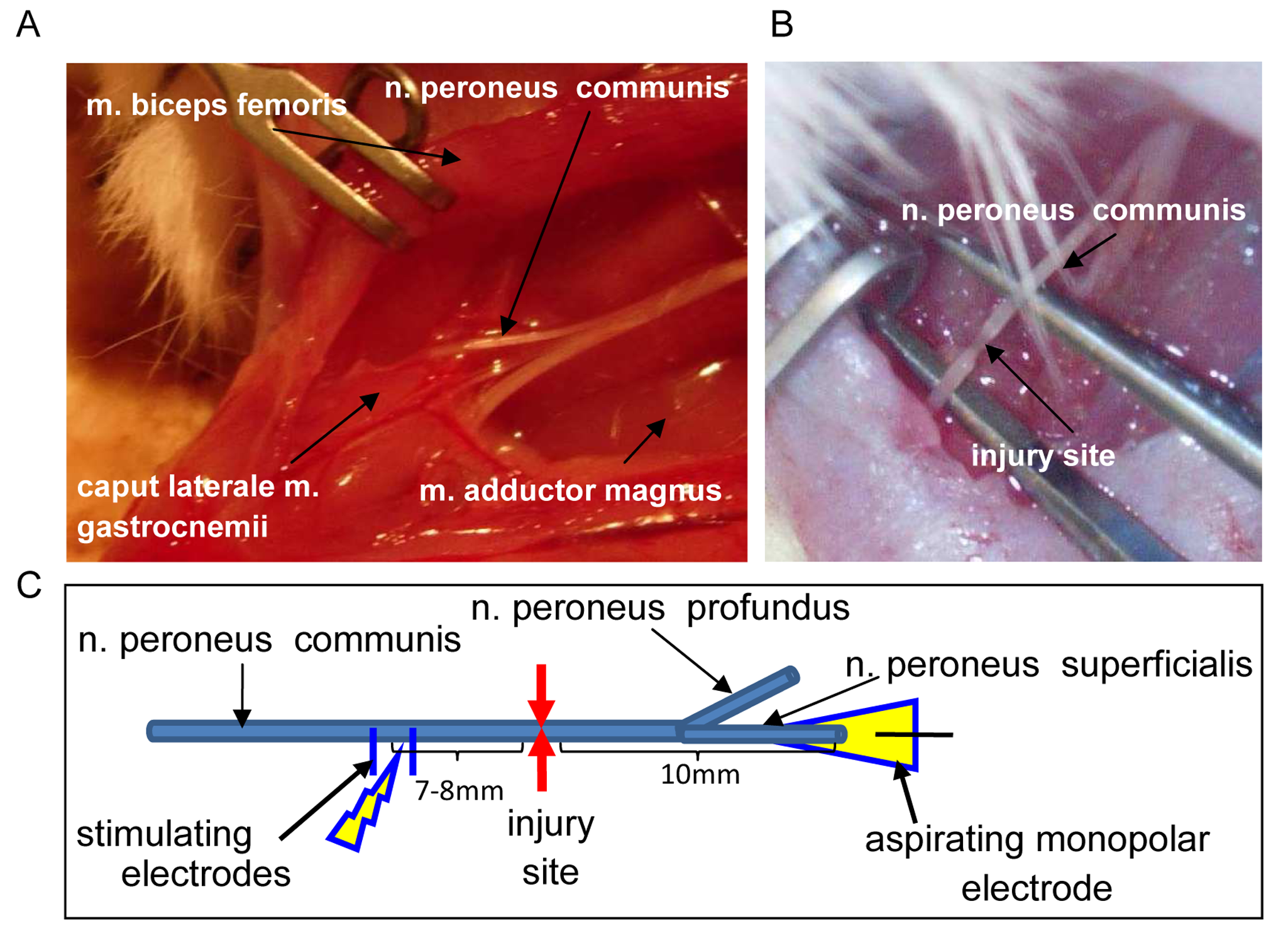

Supplement: Figure S1 — Design of nerve injury model. A – photography of n. peroneus communis separated from surrounding tissue before injury. B - photography of n. peroneus communis injury site. C. - scheme of nerve conduction velocity assessment. (TIF) [file pone.0017899.s001.tif]

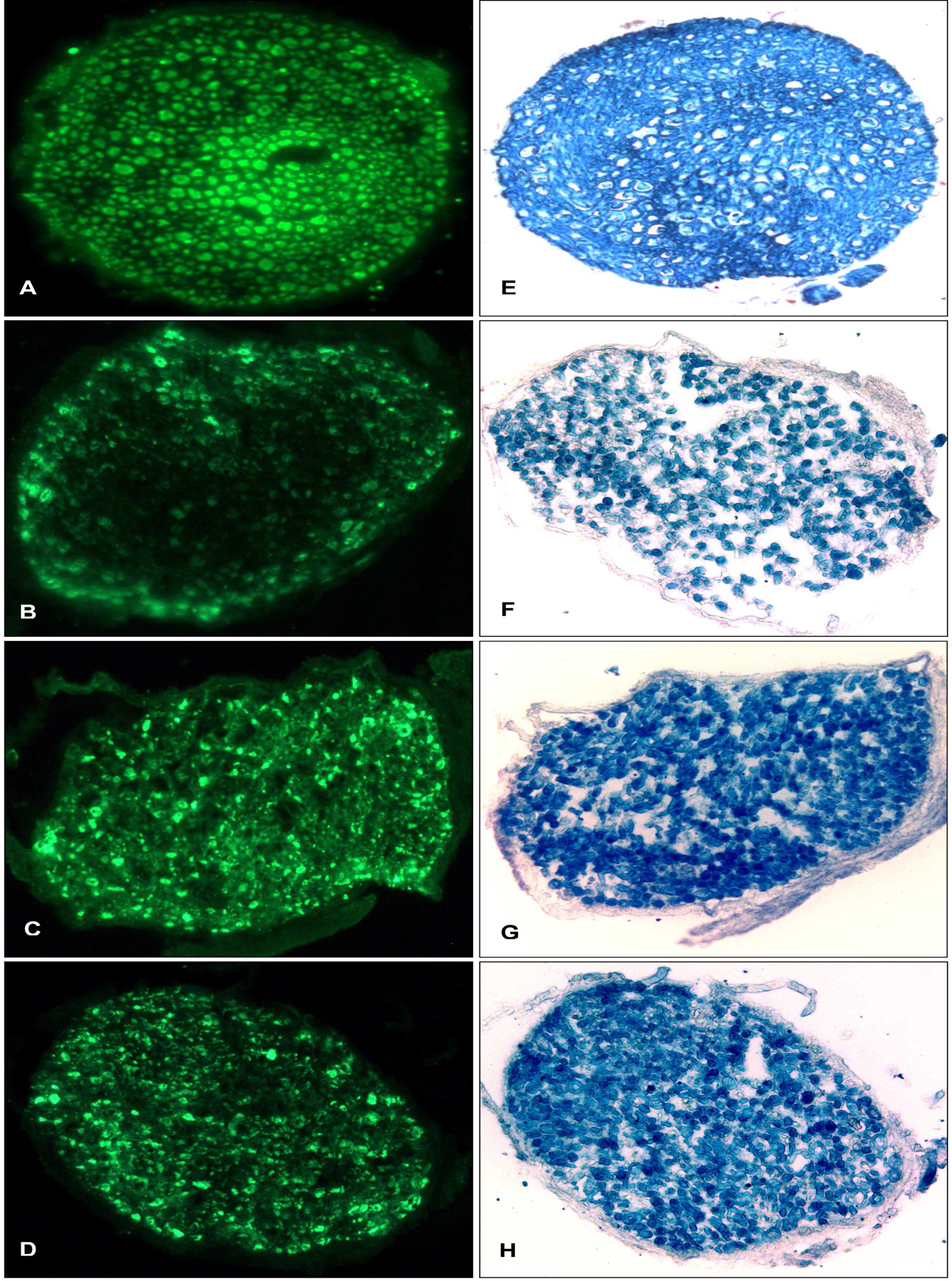

Supplement: Figure S2 — Common peroneal nerve healing 7 days after injury. A–D. - Immunofluorescent staining of frozen sections with NF-200 antibody (green fluorescence). E–H. - Staining of frozen sections with Sudan black (blue color). A, E. – uninjured nerve. B, F. – injured nerve treated with matrigel only (no cells; negative control). C, G. – injured nerve treated with mASCs. D, H. - injured nerve with matrigel only but administered 65 µg/kg vitamin B12 i.p. (positive control). (TIF) [file pone.0017899.s002.tif]
