# Supplementary material for: Adipose-Derived Stem Cells Stimulate Regeneration of Peripheral Nerves: BDNF Secreted by These Cells Promotes Nerve Healing and Axon Growth De Novo
Source: PLoS One. 2011 Mar 14;6(3):e17899. doi: 10.1371/journal.pone.0017899 (PMC3056777; doi:10.1371/journal.pone.0017899)
Supplement: Table S1 — (DOC) [file pone.0017899.s003.doc]

**Supplementary t**able. Expression of selected neurogenesis-related genes in ASCs.

| **TargetID** | **DEFINITION** |
| --- | --- |
| ALCAM | activated leukocyte cell adhesion molecule (ALCAM), mRNA. |
| AKT1S1 | AKT1 substrate 1 (proline-rich) (AKT1S1), mRNA. |
| AMPH | amphiphysin (AMPH), transcript variant 1, mRNA. |
| APOE | apolipoprotein E (APOE), mRNA. |
| ASPM | asp (abnormal spindle) homolog, microcephaly associated, mRNA. |
| BMP4 | bone morphogenetic protein 4 (BMP4), transcript variant 3, mRNA. |
| BEX2 | brain expressed X-linked 2 (BEX2), mRNA. |
| BRP44 | brain protein 44 (BRP44), mRNA. |
| BRP44L | brain protein 44-like (BRP44L), mRNA. |
| BRI3 | brain protein I3 (BRI3), mRNA. |
| BDNF | brain-derived neurotrophic factor (BDNF), transcript variant 4, mRNA. |
| CD97 | CD97 molecule (CD97), transcript variant 2, mRNA. |
| CDK5RAP3 | CDK5 regulatory subunit associated protein 3 (CDK5RAP3), mRNA. |
| CGNL1 | cingulin-like 1 (CGNL1), mRNA. |
| CLDN12 | claudin 12 (CLDN12), mRNA. |
| DLGAP2 | discs, large (Drosophila) homolog-associated protein 2 (DLGAP2), mRNA. |
| DLG4 | discs, large homolog 4 (Drosophila) (DLG4), mRNA. |
| DCLK1 | doublecortin-like kinase 1 (DCLK1), mRNA. |
| DBN1 | drebrin 1 (DBN1), transcript variant 1, mRNA. |
| FARP2 | FERM, RhoGEF and pleckstrin domain protein 2 (FARP2), mRNA. |
| GAL | galanin prepropeptide (GAL), mRNA. |
| GLS | glutaminase (GLS), mRNA. |
| EGR2 | early growth response 2 (Krox-20 homolog, Drosophila) (EGR2), mRNA. |
| KATNA1 | katanin p60 (ATPase-containing) subunit A 1 (KATNA1), mRNA. |
| LAMA1 | laminin, alpha 1 (LAMA1), mRNA. |
| LAMA2 | laminin, alpha 2 (LAMA2), transcript variant 1, mRNA. |
| LAMA2 | laminin, alpha 2 (LAMA2), transcript variant 2, mRNA. |
| LAMA3 | laminin, alpha 3 (LAMA3), transcript variant 1, mRNA. |
| LAMB2 | laminin, beta 2 (laminin S) (LAMB2), mRNA. |
| MDGA1 | MAM domain containing glycosylphosphatidylinositol anchor 1 (MDGA1), mRNA. |
| MTDH | metadherin (MTDH), mRNA. |
| MAP1S | microtubule-associated protein 1S (MAP1S), mRNA. |
| MBP | myelin basic protein (MBP), transcript variant 3, mRNA. |
| MBP | myelin basic protein (MBP), transcript variant 7, mRNA. |
| MBP | myelin basic protein (MBP), transcript variant 8, mRNA. |
| MPZL1 | myelin protein zero-like 1 (MPZL1), transcript variant 1, mRNA. |
| MPZL1 | myelin protein zero-like 1 (MPZL1), transcript variant 2, mRNA. |
| MPZL2 | myelin protein zero-like 2 (MPZL2), transcript variant 1, mRNA. |
| NDN | necdin homolog (mouse) (NDN), mRNA. |
| NGF | nerve growth factor (beta polypeptide) (NGF), mRNA. |
| NES | nestin (NES), mRNA. |
| NTN4 | netrin 4 (NTN4), mRNA. |
| NTNG1 | netrin G1 (NTNG1), transcript variant 3, mRNA. |
| NCAM1 | neural cell adhesion molecule 1 (NCAM1), transcript variant 1, mRNA. |
| NCAM2 | neural cell adhesion molecule 2 (NCAM2), mRNA. |
| NPDC1 | neural proliferation, differentiation and control, 1 (NPDC1), mRNA. |
| NXPH4 | neurexophilin 4 (NXPH4), mRNA. |
| NF1 | neurofibromin 1 (NF1), transcript variant 1, mRNA. |
| NF2 | neurofibromin 2 (bilateral acoustic neuroma) (NF2), transcript variant 1, mRNA. |
| NEFM | neurofilament, medium polypeptide 150kDa (NEFM), mRNA. |
| NETO2 | neuropilin (NRP) and tolloid (TLL)-like 2 (NETO2), mRNA. |
| NPTN | neuroplastin (NPTN), transcript variant beta, mRNA. |
| NOTCH1 | Notch homolog 1, translocation-associated (Drosophila) (NOTCH1), mRNA. |
| NINJ2 | ninjurin 2 (NINJ2), mRNA. |
| NRG1 | neuregulin 1 (NRG1), transcript variant GGF2, mRNA. |
| PRX | periaxin (PRX), transcript variant 1, mRNA. |
| PMP22 | peripheral myelin protein 22 (PMP22), transcript variant 1, mRNA. |
| PMP22 | peripheral myelin protein 22 (PMP22), transcript variant 2, mRNA. |
| PSEN1 | presenilin 1 (Alzheimer disease 3) (PSEN1), mRNA. |
| PENK | proenkephalin (PENK), mRNA. |
| PLP1 | proteolipid protein 1 (PLP1), transcript variant 2, mRNA. |
| PCDH18 | protocadherin 18 (PCDH18), mRNA. |
| PCDH24 | protocadherin 24 (PCDH24), mRNA. |
| PCDH7 | protocadherin 7 (PCDH7), transcript variant a, mRNA. |
| RAPSN | receptor-associated protein of the synapse (RAPSN), mRNA. |
| RTN1 | reticulon 1 (RTN1), transcript variant 1, mRNA. |
| SLITRK4 | SLIT and NTRK-like family, member 4 (SLITRK4), mRNA. |
| SLITRK4 | SLIT and NTRK-like family, member 4 (SLITRK4), mRNA. |
| SLIT2 | slit homolog 2 (Drosophila) (SLIT2), mRNA. |
| SLIT3 | slit homolog 3 (Drosophila) (SLIT3), mRNA. |
| SYMPK | symplekin (SYMPK), mRNA. |
| SYN1 | synapsin I (SYN1), transcript variant Ia, mRNA. |
| SYN1 | synapsin I (SYN1), transcript variant Ia, mRNA. |
| SYNGR1 | synaptogyrin 1 (SYNGR1), transcript variant 1a, mRNA. |
| SYPL1 | synaptophysin-like 1 (SYPL1), transcript variant 2, mRNA. |
| SYNPO2L | synaptopodin 2-like (SYNPO2L), mRNA. |
| SNAP23 | synaptosomal-associated protein, 23kDa (SNAP23), transcript variant 1, mRNA. |
| SNAP29 | synaptosomal-associated protein, 29kDa (SNAP29), mRNA. |
| SYNCRIP | synaptotagmin binding, cytoplasmic RNA interacting protein (SYNCRIP), mRNA. |
| SYNC1 | syncoilin, intermediate filament 1 (SYNC1), mRNA. |
| SNPH | syntaphilin (SNPH), mRNA. |
| STX1A | syntaxin 1A (brain) (STX1A), mRNA. |
| SNCAIP | synuclein, alpha interacting protein (SNCAIP), mRNA. |
| TJAP1 | tight junction associated protein 1 (peripheral) (TJAP1), mRNA. |
| TUBB3 | tubulin, beta 3 (TUBB3), mRNA. |
| VAMP1 | vesicle-associated membrane protein 1 (synaptobrevin 1) (VAMP1), transcript variant 2, mRNA. |
| VAMP2 | vesicle-associated membrane protein 2 (synaptobrevin 2) (VAMP2), mRNA. |
| VGF | VGF nerve growth factor inducible (VGF), mRNA. |
